# Crystal structure, Hirshfeld surface analysis and DFT studies of 6-[(*E*)-2-(thio­phen-2-yl)ethenyl]-4,5-di­hydro­pyridazin-3(2*H*)-one

**DOI:** 10.1107/S2056989019015147

**Published:** 2019-11-15

**Authors:** Said Daoui, Emine Berrin Çınar, Fouad El Kalai, Rafik Saddik, Necmi Dege, Khalid Karrouchi, Noureddine Benchat

**Affiliations:** a Laboratory of Applied Chemistry and Environment (LCAE), Faculty of Sciences, Mohamed I University, 60000 Oujda, Morocco; b Department of Physics, Faculty of Arts and Sciences, Ondokuz Mayıs, University, Samsun, Turkey; c Laboratory of Organic Synthesis, Extraction and Valorization, Faculty of Sciences, Ain Chok, University Hassan II, Casablanca, Rabat, Morocco; d Laboratory of Plant Chemistry, Organic and Bioorganic Synthesis, URAC23, Faculty of Science, BP 1014, GEOPAC Research Center, Mohammed V University, Rabat, Morocco

**Keywords:** crystal structure, di­hydro­pyridazine, DFT, mol­ecular electrostatic potential, pyridazine, thio­phen

## Abstract

In the title compound, C_10_H_10_N_2_OS, the five atoms of the thio­phene ring are essentially coplanar (r.m.s. deviation = 0.0037 Å) and the pyridazine ring is non-planar. In the crystal, pairs of N—H⋯O hydrogen bonds link the mol­ecules into dimers with an *R*_2_^2^(8) ring motif. The dimers are linked by C—H⋯O inter­actions, forming layers parallel to the *bc* plane. The theoretical geometric parameters are in good agreement with XRD results. The inter­molecular inter­actions were investigated using a Hirshfeld surface analysis and two-dimensional fingerprint plots. The Hirshfeld surface analysis of the title compound suggests that the most significant contributions to the crystal packing are by H⋯H (39.7%), C⋯H/H⋯C (17.3%) and O⋯H/H⋯O (16.8%) contacts.

## Abstract 2

In the crystal, the mol­ecules are linked by N—H⋯O and C—H⋯O inter­actions, forming a three-dimensional network. The theoretical geometrical parameters are in good agreement with XRD results.

## Chemical context   

**Figure 1:**
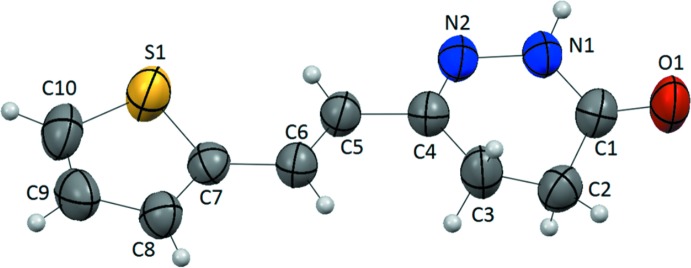
Mol­ecular structure of the title compound showing the atom labelling and displacement ellipsoids drawn at the 50% probability level.

Pyridazinone derivatives have been tested for their chemical and biological properties and achieved an increased inter­est in recent years (Akhtar *et al.*, 2016 ▸). The pyridazinone moiety is known as a ‘wonder nucleus’ as it can form diverse derivatives with many types of pharmacological activities such as anti­depressant (Boukharsa *et al.*, 2016 ▸), anti-HIV (Livermore *et al.*, 1993 ▸), anti-inflammatory (Barberot *et al.*, 2018 ▸), anti­convulsant (Partap *et al.*, 2018 ▸), anti­histaminic (Tao *et al.* 2012 ▸) and glucan synthase inhibition (Zhou *et al.*, 2011 ▸) as well as acting as herbicidal agents (Asif, 2013 ▸). We report the synthesis and the crystal and mol­ecular structure of the title compound (Fig. 1 ▸), as well as an analysis of its Hirshfeld surface and DFT studies.
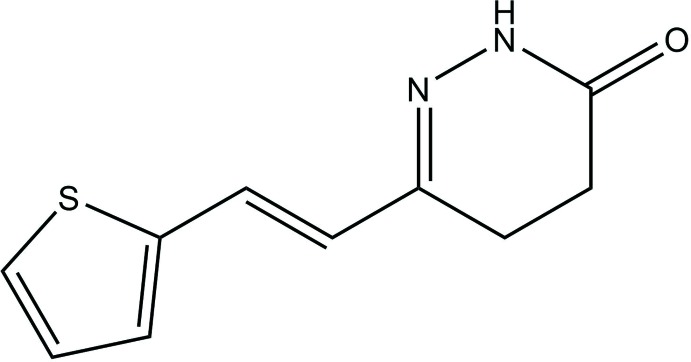


## Structural commentary   

Selected geometrical parameters are given in Table 1 ▸. The five atoms of the thio­phene ring are essentially coplanar (r.m.s. deviation = 0.0037 Å) while the pyridazine ring is non-planar with atom C2 furthest from the mean mol­ecular plane at a distance of 0.610 (5) Å.

## Supra­molecular features   

**Figure 2:**
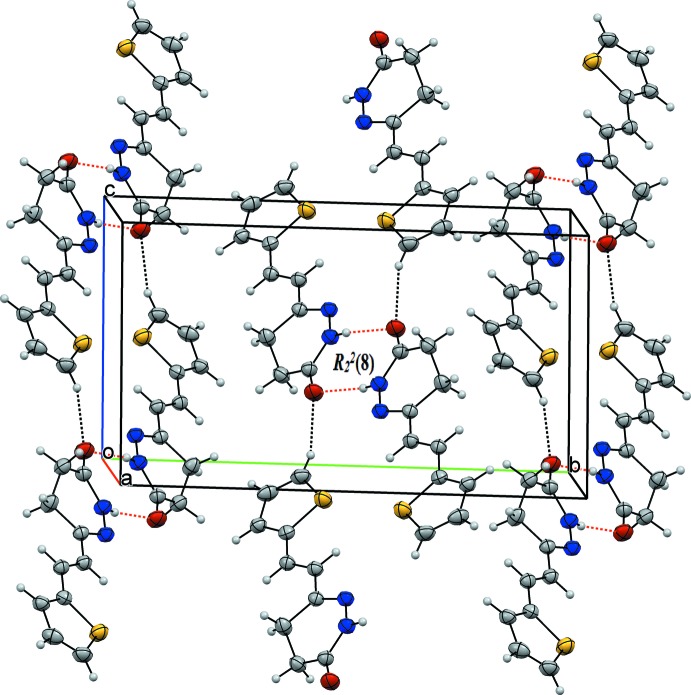
Inversion dimers with 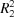
(8) ring motifs formed by N—H⋯O hydrogen bonds (red dashed lines; Table 2 ▸). C—H⋯O inter­actions are shown as black dashed lines.

In the crystal, the mol­ecules are connected pairwise through N—H⋯O hydrogen bonds (Table 2 ▸), forming dimers with an 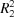
(8) graph set motif. The dimers are linked by C—H⋯O hydrogen bonds, forming layers parallel to the *bc* plane (Fig. 2 ▸). A packing diagram is shown in Fig. 2 ▸.

## Database survey   

A search of the Cambridge Structural Database (CSD, version 5.40, update November 2018; Groom *et al.*, 2016 ▸) using (*E*)-6-(thio­phen-2-yl)hex-5-enal and 6-vinyl-4,5-di­hydro­pyridazin-3(2*H*)-one as the main skeleton found two structures similar to the title compound containing the pyridazine moiety with different substituents: 4-chloro-2-[(5-eth­oxy-1,3,4-thia­diazol-2-yl)meth­yl]-5-(piperidin-1-yl)pyridazin-3(2*H*)-one (DOP­ZAL; Li *et al.*, 2014 ▸) and 4-[(*tert*-butyl­diphenyl­sil­yloxy)meth­yl]pyridazin-3(2*H*)-one (CISPAX; Costas-Lago *et al.*, 2013 ▸). In DOPZAL, the six atoms of the 1,6-di­hydro­pyridazine ring are essentially coplanar (r.m.s. deviation = 0.008 Å), and the dihedral angle between this and the 1,3,4-thia­diazole ring is 62.06 (10)°. In CISPAX, pyridazinone moieties are anti-oriented across the Si—O bond [torsion angle = 168.44 (19)°]. In the crystal, mol­ecules are assembled into inversion dimers through co-operative N—H⋯O hydrogen bonds between the NH groups and O atoms of the pyridazinone rings of neighbouring mol­ecules.

## Surface Analysis (SA)   

**Figure 3:**
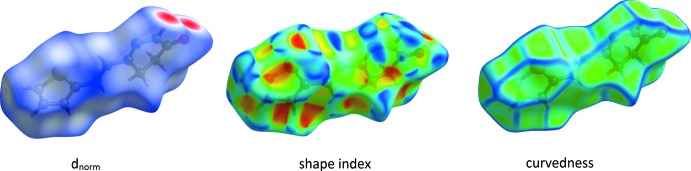
The Hirshfeld surfaces of the title compound mapped over *d*_norm_, shape-index and curvedness.

**Figure 4:**
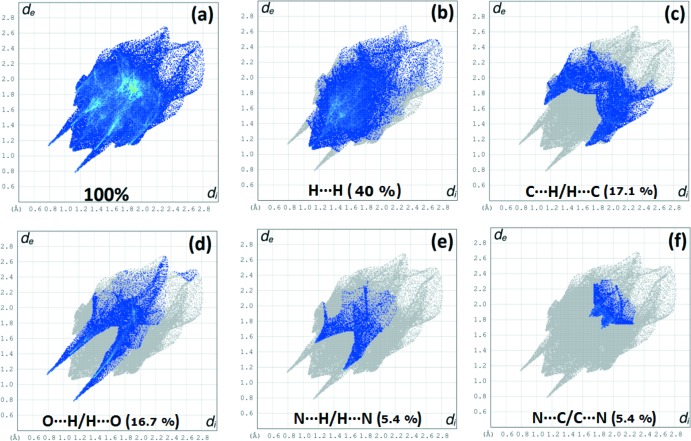
Two-dimensional fingerprint plots for the title compound, with a *d*_norm_ view and the relative contributions of the atom pairs to the Hirshfeld surface.

The Hirshfeld surface analysis (Spackman & Jayatilaka, 2009 ▸) and the associated fingerprint plots were performed with *CrystalExplorer17.5* (Turner *et al.*, 2017 ▸). This software was used to analyse the inter­molecular inter­actions in the crystal and to generate fingerprint plots mapped over *d*_norm_, shape index and curvedness (Fig. 3 ▸). The Hirshfeld surface was calculated using a standard (high) surface resolution with the three-dimensional *d*_norm_ surface plotted over a fixed colour scale of −0.532 (red) to 1.345 (blue) a.u. The pale-red spots symbolize short contacts and negative *d*_norm_ values on the surface correspond to the N—H⋯O and C—H⋯O inter­actions (Table 2 ▸). The overall fingerprint plot and those delin­eated into H⋯H, H⋯C/ C⋯H, H⋯O/O⋯H, N⋯H/H⋯N and N⋯·C/C⋯·N contacts are shown in Fig. 4 ▸ along with their relative contributions to the Hirshfeld surface. The largest contribution is from H⋯H inter­actions (40.0%). The shape-index map of the title complex was generated in the range −1 to 1 Å, with the convex blue regions indicating hydrogen-donor groups and the concave red regions hydrogen-acceptor groups. The curvedness map, generated in the range −4 to 0.4 Å, shows large regions of green which denote a relatively flat surface area (planar), while the blue regions denote areas of curvature.

**Figure 5:**
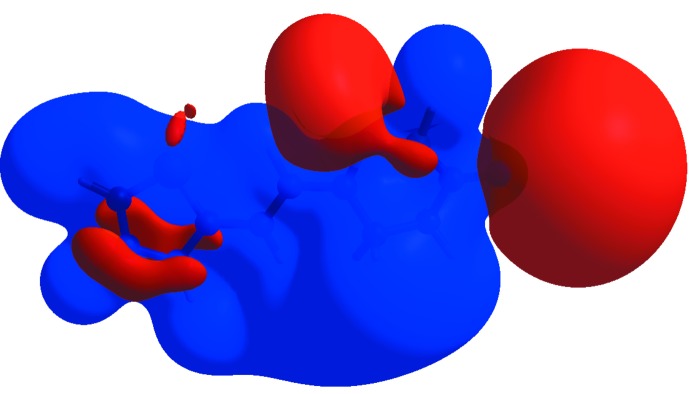
A view of the mol­ecular electrostatic potential for the title compound in the range −0.084 to 0.084 a.u. generated by DFT using the 6–31G(d,p) basis set.

A view of the mol­ecular electrostatic potential, in the range − 0.084 to 0.084 a.u. generated by the DFT method using the 6-31G(d,p) basis set is shown in Fig. 5 ▸. Here the N—H⋯O hydrogen-bond donors and acceptors are shown as blue and red areas around the atoms related with positive (hydrogen-bond donors) and negative (hydrogen-bond acceptors) electrostatic potentials, respectively.

The theoretical calculations were performed using *GAUSSIAN03* (Frisch *et al.*, 2004 ▸). The initial geometry was taken from the X-ray coordinates and this geometry was optimized using the DFT/B3LYP (Becke, 1993 ▸) method with LANL2DZ as the basis set. The theoretical geometrical parameters are in good agreement with XRD results (Table 1 ▸).

## Frontier mol­ecular orbitals   

**Figure 6:**
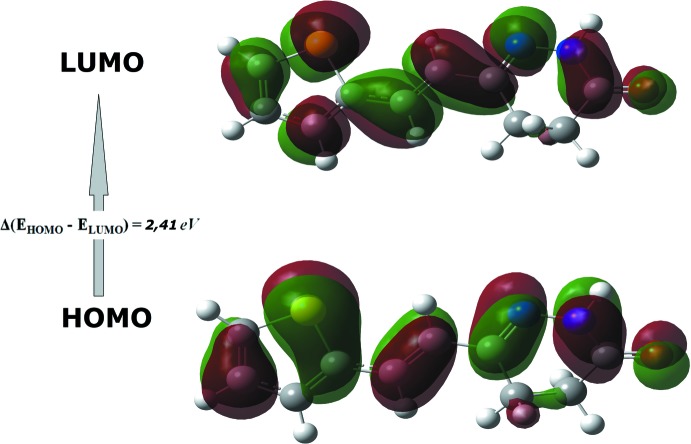
The electron distribution of the HOMO and LUMO energy gaps of the title compound.

**Figure 7:**
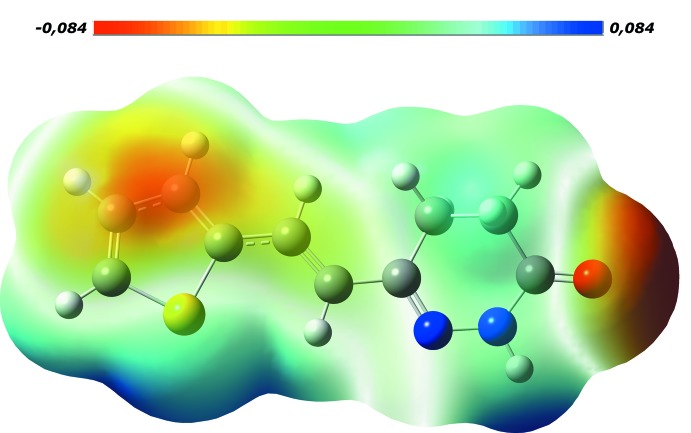
The total electron density three-dimensional surface mapped for the compound with the electrostatic potential calculated at the B3LYP/6–31G(d,p) level.

The highest occupied mol­ecular orbitals (HOMO) and the lowest unoccupied mol­ecular orbitals (LUMO) are known as frontier mol­ecular orbitals (FMOs). The FMOs play an important role in the optical and electric properties. The frontier orbital gap can indicate the chemical reactivity and the kinetic stability of the mol­ecule. If the energy gap is small then the mol­ecule is highly polarizable and has high chemical reactivity. A mol­ecule with a small frontier orbital gap is generally associated with a high chemical reactivity, low kinetic stability and is termed a soft mol­ecule. Fig. 6 ▸ illustrates the HOMO and LUMO energy levels of the title compound. The small HOMO–LUMO energy gap of 2.41 eV in this compound indicates the chemical reactivity is strong and the kinetic stability is weak. A map of the electron density is shown in Fig. 7 ▸.

## Synthesis and crystallization   

To a solution of 4-oxo-6-(thio­phen-2-yl)hex-5-enoic acid (0.21 g, 1 mmol) in 20 mL of ethanol, it was added an equimolar amount of hydrazine hydrate. The mixture was maintained under reflux for 4h, until TLC indicated the end of the reaction. The reaction mixture was poured into cold water, and the precipitate formed was filtered out, washed with ethanol and recrystallized from ethanol. Slow evaporation at room temperature led to formation of single crystals.

## Refinement   

Crystal data, data collection and structure refinement details are summarized in Table 3 ▸. Hydrogen atoms were fixed geometrically and treated as riding, the C-bound H atoms were placed in idealized positions and refined as riding: C—H = 0.93 Å for methyl­ene *U*_iso_(H) = 1.5*U*_eq_(C) and C—H = 0.97 Å for the other C atoms with *U*_iso_(H) = 1.2*U*_eq_(C). The NH H atom was located in a difference-Fourier map and freely refined.

## Supplementary Material

Crystal structure: contains datablock(s) I. DOI: 10.1107/S2056989019015147/mw2150sup1.cif

Structure factors: contains datablock(s) I. DOI: 10.1107/S2056989019015147/mw2150Isup2.hkl

CCDC references: 1964913, 1964910, 1964910

Additional supporting information:  crystallographic information; 3D view; checkCIF report

## Figures and Tables

**Table 1:** Selected bond lengths, angles and torsion angles (Å, °)

	X-ray	DFT/B3LYP/LANL2DZ
S1—C10	1.691 (3)	1.734 (7)
O1—C1	1.227 (3)	1.219 (9)
N2—C4	1.283 (3)	1.300 (2)
N2—N1	1.385 (2)	1.375 (6)
N1—C1	1.345 (3)	1.353 (0)
		
C10—S1—C7	92.31 (13)	91.705 (6)
O1—C1—N1	121.4 (2)	121.412 (2)
		
C7—C6—C5—C4	179.0 (2)	178.857 (8)
N2—C4—C5—C6	−179.4 (2)	179.554 (8)

**Table 2:** Hydrogen-bond geometry (Å, °)

*D*—H⋯*A*	*D*—H	H⋯*A*	*D*⋯*A*	*D*—H⋯*A*
N1—H1⋯O1^i^	0.83 (3)	2.07 (3)	2.899 (3)	175 (2)
C10—H10⋯O1^ii^	0.93	2.61	3.505 (3)	161

Symmetry codes: (i) 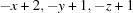; (ii) 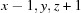.

**Table 3:** Experimental details

Crystal data	
Chemical formula	C_10_H_10_N_2_OS
*M*_r_	206.26
Crystal system, space group	Monoclinic, *P*2_1_/*c*
Temperature (K)	296
*a*, *b*, *c* (Å)	6.9932 (5), 16.2916 (9), 9.3544 (7)
β (°)	110.168 (6)
*V* (Å^3^)	1000.40 (12)
*Z*	4
Radiation type	Mo *K*α
μ (mm^−1^)	0.29
Crystal size (mm)	0.78 × 0.43 × 0.25

Data collection	
Diffractometer	Stoe *IPDS* 2
Absorption correction	Integration (*X-RED32*; Stoe & Cie, 2002 ▸)
*T*_min_, *T*_max_	0.767, 0.932
No. of measured, independent and observed [*I* > 2σ(*I*)] reflections	6292, 1971, 1394
*R*_int_	0.043
(sin θ/λ)_max_ (Å^−1^)	0.617

Refinement	
*R*[*F* ^2^ > 2σ(*F* ^2^)], *wR*(*F* ^2^), *S*	0.045, 0.135, 1.02
No. of reflections	1971
No. of parameters	131
No. of restraints	19
H-atom treatment	H atoms treated by a mixture of independent and constrained refinement
Δρ_max_, Δρ_min_ (e Å^−3^)	0.24, −0.34

Computer programs: *X-AREA* and *X-RED32* (Stoe & Cie, 2002 ▸), *SHELXT2015* (Sheldrick, 2015*a* ▸), *SHELXL2015* (Sheldrick, 2015*b* ▸), *Mercury* (Macrae *et al.*, 2008 ▸), *WinGX* (Farrugia, 2012 ▸) and *PLATON* (Spek, 2009 ▸).

## supplementary crystallographic information

### Crystal data

**Table:**

C_10_H_10_N_2_OS	*F*(000) = 432
*M**_r_* = 206.26	*D*_x_ = 1.369 Mg m^−^^3^
Monoclinic, *P*2_1_/*c*	Mo *K*α radiation, λ = 0.71073 Å
*a* = 6.9932 (5) Å	Cell parameters from 6992 reflections
*b* = 16.2916 (9) Å	θ = 2.3–30.0°
*c* = 9.3544 (7) Å	µ = 0.29 mm^−^^1^
β = 110.168 (6)°	*T* = 296 K
*V* = 1000.40 (12) Å^3^	Stick, orange
*Z* = 4	0.78 × 0.43 × 0.25 mm

### Data collection

**Table:**

Stoe IPDS 2 diffractometer	1971 independent reflections
Radiation source: sealed X-ray tube, 12 x 0.4 mm long-fine focus	1394 reflections with *I* > 2σ(*I*)
Plane graphite monochromator	*R*_int_ = 0.043
Detector resolution: 6.67 pixels mm^-1^	θ_max_ = 26.0°, θ_min_ = 2.5°
rotation method scans	*h* = −8→8
Absorption correction: integration (X-RED32; Stoe & Cie, 2002)	*k* = −20→19
*T*_min_ = 0.767, *T*_max_ = 0.932	*l* = −11→11
6292 measured reflections	

### Refinement

**Table:**

Refinement on *F*^2^	19 restraints
Least-squares matrix: full	Hydrogen site location: mixed
*R*[*F*^2^ > 2σ(*F*^2^)] = 0.045	H atoms treated by a mixture of independent and constrained refinement
*wR*(*F*^2^) = 0.135	*w* = 1/[σ^2^(*F*_o_^2^) + (0.079*P*)^2^] where *P* = (*F*_o_^2^ + 2*F*_c_^2^)/3
*S* = 1.02	(Δ/σ)_max_ < 0.001
1971 reflections	Δρ_max_ = 0.24 e Å^−^^3^
131 parameters	Δρ_min_ = −0.34 e Å^−^^3^

### Special details

**Table:**

Geometry. All esds (except the esd in the dihedral angle between two l.s. planes) are estimated using the full covariance matrix. The cell esds are taken into account individually in the estimation of esds in distances, angles and torsion angles; correlations between esds in cell parameters are only used when they are defined by crystal symmetry. An approximate (isotropic) treatment of cell esds is used for estimating esds involving l.s. planes.

### Fractional atomic coordinates and isotropic or equivalent isotropic displacement parameters (Å^2^)

**Table:**

	*x*	*y*	*z*	*U*_iso_*/*U*_eq_	
S1	0.23031 (12)	0.42526 (4)	0.98925 (7)	0.0768 (3)	
O1	0.8844 (3)	0.41724 (11)	0.36257 (19)	0.0752 (5)	
N2	0.6910 (3)	0.45665 (12)	0.66000 (19)	0.0559 (5)	
N1	0.8097 (3)	0.45451 (13)	0.5682 (2)	0.0581 (5)	
C4	0.5618 (3)	0.39839 (14)	0.6440 (2)	0.0521 (5)	
C7	0.1949 (3)	0.34895 (13)	0.8563 (2)	0.0517 (5)	
C6	0.3065 (3)	0.34666 (13)	0.7517 (2)	0.0535 (5)	
H6	0.277544	0.303662	0.682065	0.064*	
C5	0.4468 (3)	0.40054 (14)	0.7461 (2)	0.0549 (5)	
H5	0.473561	0.443933	0.814790	0.066*	
C8	0.0484 (4)	0.29384 (15)	0.8660 (2)	0.0607 (6)	
H8	0.005051	0.248100	0.803758	0.073*	
C1	0.7734 (3)	0.41133 (14)	0.4389 (2)	0.0568 (6)	
C2	0.5880 (4)	0.35925 (15)	0.3960 (2)	0.0640 (6)	
H2A	0.607918	0.311934	0.339710	0.077*	
H2B	0.473610	0.390273	0.329261	0.077*	
C3	0.5376 (4)	0.33001 (16)	0.5325 (3)	0.0681 (6)	
H3A	0.398564	0.309944	0.499271	0.082*	
H3B	0.627316	0.285060	0.581438	0.082*	
C10	0.0570 (4)	0.38383 (19)	1.0572 (3)	0.0739 (7)	
H10	0.023862	0.405884	1.137400	0.089*	
C9	−0.0260 (4)	0.31634 (18)	0.9828 (3)	0.0713 (6)	
H9	−0.124891	0.286306	1.005625	0.086*	
H1	0.902 (4)	0.4897 (15)	0.593 (2)	0.058 (7)*	

### Atomic displacement parameters (Å^2^)

**Table:**

	*U*^11^	*U*^22^	*U*^33^	*U*^12^	*U*^13^	*U*^23^
S1	0.0979 (5)	0.0736 (5)	0.0729 (4)	−0.0147 (4)	0.0476 (4)	−0.0139 (3)
O1	0.0895 (12)	0.0816 (12)	0.0746 (10)	−0.0144 (10)	0.0540 (9)	−0.0103 (8)
N2	0.0594 (11)	0.0619 (11)	0.0527 (9)	−0.0040 (9)	0.0272 (8)	−0.0029 (8)
N1	0.0604 (11)	0.0644 (12)	0.0580 (10)	−0.0108 (10)	0.0312 (9)	−0.0065 (9)
C4	0.0534 (11)	0.0535 (12)	0.0522 (11)	0.0012 (10)	0.0216 (9)	−0.0007 (9)
C7	0.0521 (11)	0.0561 (13)	0.0465 (10)	0.0032 (10)	0.0165 (9)	0.0054 (9)
C6	0.0573 (12)	0.0530 (13)	0.0542 (11)	0.0002 (10)	0.0242 (10)	−0.0016 (9)
C5	0.0610 (12)	0.0565 (13)	0.0525 (11)	−0.0034 (11)	0.0261 (9)	−0.0043 (9)
C8	0.0651 (13)	0.0659 (14)	0.0543 (11)	−0.0087 (11)	0.0249 (9)	0.0049 (9)
C1	0.0655 (13)	0.0554 (13)	0.0550 (11)	0.0023 (11)	0.0279 (10)	0.0014 (9)
C2	0.0696 (14)	0.0668 (15)	0.0599 (12)	−0.0052 (12)	0.0281 (11)	−0.0118 (10)
C3	0.0757 (15)	0.0639 (15)	0.0798 (15)	−0.0133 (12)	0.0459 (13)	−0.0150 (12)
C10	0.0869 (17)	0.0885 (19)	0.0583 (13)	0.0085 (16)	0.0403 (12)	0.0062 (13)
C9	0.0686 (14)	0.0910 (18)	0.0624 (12)	−0.0097 (13)	0.0329 (10)	0.0097 (12)

### Geometric parameters (Å, º)

**Table:**

S1—C10	1.691 (3)	C5—H5	0.9300
S1—C7	1.715 (2)	C8—C9	1.411 (3)
O1—C1	1.227 (3)	C8—H8	0.9300
N2—C4	1.283 (3)	C1—C2	1.484 (3)
N2—N1	1.385 (2)	C2—C3	1.515 (3)
N1—C1	1.345 (3)	C2—H2A	0.9700
N1—H1	0.83 (2)	C2—H2B	0.9700
C4—C5	1.446 (3)	C3—H3A	0.9700
C4—C3	1.495 (3)	C3—H3B	0.9700
C7—C8	1.388 (3)	C10—C9	1.323 (4)
C7—C6	1.448 (3)	C10—H10	0.9300
C6—C5	1.331 (3)	C9—H9	0.9300
C6—H6	0.9300		
			
C10—S1—C7	92.31 (13)	O1—C1—C2	123.8 (2)
C4—N2—N1	117.21 (18)	N1—C1—C2	114.77 (19)
C1—N1—N2	127.1 (2)	C1—C2—C3	112.76 (18)
C1—N1—H1	119.6 (16)	C1—C2—H2A	109.0
N2—N1—H1	112.7 (16)	C3—C2—H2A	109.0
N2—C4—C5	115.76 (19)	C1—C2—H2B	109.0
N2—C4—C3	122.57 (19)	C3—C2—H2B	109.0
C5—C4—C3	121.6 (2)	H2A—C2—H2B	107.8
C8—C7—C6	127.6 (2)	C4—C3—C2	110.5 (2)
C8—C7—S1	110.28 (16)	C4—C3—H3A	109.5
C6—C7—S1	122.15 (16)	C2—C3—H3A	109.5
C5—C6—C7	125.9 (2)	C4—C3—H3B	109.5
C5—C6—H6	117.1	C2—C3—H3B	109.5
C7—C6—H6	117.1	H3A—C3—H3B	108.1
C6—C5—C4	126.5 (2)	C9—C10—S1	112.02 (19)
C6—C5—H5	116.7	C9—C10—H10	124.0
C4—C5—H5	116.7	S1—C10—H10	124.0
C7—C8—C9	111.1 (2)	C10—C9—C8	114.3 (2)
C7—C8—H8	124.4	C10—C9—H9	122.9
C9—C8—H8	124.4	C8—C9—H9	122.9
O1—C1—N1	121.4 (2)		
			
C4—N2—N1—C1	18.1 (3)	S1—C7—C8—C9	−0.6 (2)
N1—N2—C4—C5	177.40 (18)	N2—N1—C1—O1	176.4 (2)
N1—N2—C4—C3	0.3 (3)	N2—N1—C1—C2	−1.9 (3)
C10—S1—C7—C8	0.74 (18)	O1—C1—C2—C3	152.4 (2)
C10—S1—C7—C6	−179.65 (18)	N1—C1—C2—C3	−29.5 (3)
C8—C7—C6—C5	−179.7 (2)	N2—C4—C3—C2	−30.2 (3)
S1—C7—C6—C5	0.8 (3)	C5—C4—C3—C2	152.9 (2)
C7—C6—C5—C4	179.0 (2)	C1—C2—C3—C4	43.3 (3)
N2—C4—C5—C6	−179.4 (2)	C7—S1—C10—C9	−0.7 (2)
C3—C4—C5—C6	−2.3 (4)	S1—C10—C9—C8	0.5 (3)
C6—C7—C8—C9	179.8 (2)	C7—C8—C9—C10	0.1 (3)

### Hydrogen-bond geometry (Å, º)

**Table:**

*D*—H···*A*	*D*—H	H···*A*	*D*···*A*	*D*—H···*A*
N1—H1···O1^i^	0.83 (3)	2.07 (3)	2.899 (3)	175 (2)
C10—H10···O1^ii^	0.93	2.61	3.505 (3)	161

Symmetry codes: (i) −*x*+2, −*y*+1, −*z*+1; (ii) *x*−1, *y*, *z*+1.

## References

[bb1] Akhtar, W., Shaquiquzzaman, M., Akhter, M., Verma, G., Khan, M. F. & Alam, M. M. (2016). *Eur. J. Med. Chem.* **123**, 256–281. 10.1016/j.ejmech.2016.07.061 27484513

[bb2] Asif, M. (2013). *Mini-Rev. Org. Chem.* **10**, 113–122.

[bb3] Barberot, C., Moniot, A., Allart-Simon, I., Malleret, L., Yegorova, T., Laronze-Cochard, M., Bentaher, A., Médebielle, M., Bouillon, J., Hénon, E., Sapi, J., Velard, F. & Gérard, S. (2018). *Eur. J. Med. Chem.* **146**, 139–146. 10.1016/j.ejmech.2018.01.035 29407945

[bb4] Becke, A. D. (1993). *J. Chem. Phys.* **98**, 5648–5652.

[bb5] Boukharsa, Y., Meddah, B., Tiendrebeogo, R. Y., Ibrahimi, A., Taoufik, J., Cherrah, Y., Benomar, A., Faouzi, M. E. A. & Ansar, M. (2016). *Med. Chem. Res.* **25**, 494–500.

[bb6] Costas-Lago, M. C., Costas, T., Vila, N. & Besada, P. (2013). *Acta Cryst.* E**69**, o1859–o1860. 10.1107/S1600536813032212 PMC4004440 24860296

[bb7] Farrugia, L. J. (2012). *J. Appl. Cryst.* **45**, 849–854.

[bb8] Frisch, M. J., Trucks, G. W., Schlegel, H. B., Scuseria, G. E., Robb, M. A., Cheeseman, J. R., Montgomery, J. A. Jr, Vreven, T., Kudin, K. N., Burant, J. C., Millam, J. M., Iyengar, S. S., Tomasi, J., Barone, V., Mennucci, B., Cossi, M., Scalmani, G., Rega, N., Petersson, G. A., Nakatsuji, H., Hada, M., Ehara, M., Toyota, K., Fukuda, R., Hasegawa, J., Ishida, M., Nakajima, T., Honda, Y., Kitao, O., Nakai, H., Klene, M., Li, X., Knox, J. E., Hratchian, H. P., Cross, J. B., Bakken, V., Adamo, C., Jaramillo, J., Gomperts, R., Stratmann, R. E., Yazyev, O., Austin, A. J., Cammi, R., Pomelli, C., Ochterski, J. W., Ayala, P. Y., Morokuma, K., Voth, G. A., Salvador, P., Dannenberg, J. J., Zakrzewski, V. G., Dapprich, S., Daniels, A. D., Strain, M. C., Farkas, O., Malick, D. K., Rabuck, A. D., Raghavachari, K., Foresman, J. B., Ortiz, J. V., Cui, Q., Baboul, A. G., Clifford, S., Cioslowski, J., Stefanov, B. B., Liu, G., Liashenko, A., Piskorz, P., Komaromi, I., Martin, R. L., Fox, D. J., Keith, T., Al-Laham, M. A., Peng, C. Y., Nanayakkara, A., Challacombe, M., Gill, P. M. W., Johnson, B., Chen, W., Wong, M. W., Gonzalez, C. & Pople, J. A. (2004). *GAUSSIAN03*. Gaussian Inc., Wallingford, CT, USA.

[bb9] Groom, C. R., Bruno, I. J., Lightfoot, M. P. & Ward, S. C. (2016). *Acta Cryst.* B**72**, 171–179. 10.1107/S2052520616003954 PMC4822653 27048719

[bb10] Li, H., Ren, X., Li, Y. & Zhao, L. (2014). *Acta Cryst.* E**70**, o1113. 10.1107/S1600536814020662 PMC4257213 25484702

[bb11] Livermore, D., Bethell, R. C., Cammack, N., Hancock, A. P., Hann, M. M., Green, D., Lamont, R. B., Noble, S. A., Orr, D. C., Payne, J. P., Ramsay, M. V. J., Shingler, A. H. Smith, C., Storer, R., Williamson, C. & Willson, T. (1993). *J. Med. Chem.* **36**, 3784–3794. 10.1021/jm00076a005 7504733

[bb12] Macrae, C. F., Bruno, I. J., Chisholm, J. A., Edgington, P. R., McCabe, P., Pidcock, E., Rodriguez-Monge, L., Taylor, R., van de Streek, J. & Wood, P. A. (2008). *J. Appl. Cryst.* **41**, 466–470.

[bb13] Partap, S., Akhtar, M. J., Yar, M. S., Hassan, M. Z. & Siddiqui, A. A. (2018). *Bioorg. Chem.* **77**, 74–83. 10.1016/j.bioorg.2018.01.001 29334622

[bb14] Sheldrick, G. M. (2015*a*). *Acta Cryst.* A**71**, 3–8.

[bb15] Sheldrick, G. M. (2015*b*). *Acta Cryst.* C**71**, 3–8.

[bb16] Spackman, M. A. & Jayatilaka, D. (2009). *CrystEngComm*, **11**, 19–32.

[bb17] Spek, A. L. (2009). *Acta Cryst.* D**65**, 148–155. 10.1107/S090744490804362X PMC2631630 19171970

[bb18] Stoe & Cie (2002). *X-AREA* and *X-RED32*. Stoe & Cie GmbH, Darmstadt, Germany.

[bb19] Tao, M., Aimone, L. D., Gruner, J. A., Mathiasen, J. R., Huang, Z., Lyons, J., Raddatz, R. & Hudkins, R. L. (2012). *Bioorg. Med. Chem. Lett.* **22**, 1073–1077. 10.1016/j.bmcl.2011.11.118 22197136

[bb20] Turner, M. J., McKinnon, J. J., Wolff, S. K., Grimwood, D. J., Spackman, P. R., Jayatilaka, D. & Spackman, M. A. (2017). *Crystal Explorer17. University of Western Australia.* http://hirshfeldsurface.net.

[bb21] Zhou, G., Ting, P. C., Aslanian, R., Cao, J., Kim, D. W., Kuang, R., Lee, J. F., Schwerdt, J., Wu, H., Jason Herr, R., Zych, A. J., Yang, J., Lam, S., Wainhaus, S., Black, T. A., McNicholas, P. M., Xu, Y. & Walker, S. S. (2011). *Bioorg. Med. Chem. Lett.* **21**, 2890–2893. 10.1016/j.bmcl.2011.03.083 21489787

